# Characterization of the temperature-sensitive reaction of F_1_-ATPase by using single-molecule manipulation

**DOI:** 10.1038/srep04962

**Published:** 2014-05-14

**Authors:** Rikiya Watanabe, Hiroyuki Noji

**Affiliations:** 1 Department of Applied Chemistry, School of Engineering, The University of Tokyo, Bunkyo-ku, Tokyo 113-8656, Japan; 2 PRESTO, JST, Bunkyo-ku, Tokyo 113-8656, Japan

## Abstract

F_1_-ATPase (F_1_) is a rotary motor protein that couples ATP hydrolysis to mechanical rotation with high efficiency. In our recent study, we observed a highly temperature-sensitive (TS) step in the reaction catalyzed by a thermophilic F_1_ that was characterized by a rate constant remarkably sensitive to temperature and had a Q_10_ factor of 6–19. Since reactions with high Q_10_ values are considered to involve large conformational changes, we speculated that the TS reaction plays a key role in the rotation of F_1_. To clarify the role of the TS reaction, in this study, we conducted a stall and release experiment using magnetic tweezers, and assessed the torque generated during the TS reaction. The results indicate that the TS reaction generates the same amount of rotational torque as does ATP binding, but more than that generated during ATP hydrolysis. Thus, we confirmed that the TS reaction contributes significantly to the rotation of F_1_.

F_1_-ATPase (α_3_β_3_γδε), a catalytic subcomplex of F_o_F_1_-ATP synthase, is a rotary motor protein fuelled by ATP hydrolysis^1^,^2^,^3^,^4^. The α_3_β_3_γ subcomplex functions as the minimum component of the rotating system, in which the α_3_β_3_ subunits form a cylindrical stator and the γ rotor subunit penetrates the center of the cylinder^5^,^6^,^7^. The catalytic sites for ATP hydrolysis/synthesis are located at the interface between each α and β subunit, mainly on the β subunits; i.e., F_1_ possesses three catalytic sites. The rotary motion of F_1_ can be visualized under an optical microscope^8^,^9^,^10^. Upon ATP hydrolysis, F_1_ rotates in the anticlockwise direction (when viewed from the membrane side), in which the three β subunits cooperatively change their conformation, generating a rotational torque of 40 pN·nm rad^−1^ for the F_1_ from thermophilic *Bacillus* PS3^11^ and 20–74 pN·nm rad^−1^ for the F_1_ from *Escherichia* *coli*^10^,^12^,^13^. The energy required for the mechanical work for one γ rotation is balanced by the hydrolysis of three ATP molecules. Therefore, F_1_ is extremely efficient in converting chemical energy to mechanical work and the catalysis is tightly coupled to the mechanical work^11^,^14^.

The mechanisms underlying the catalysis and the rotation have been established^15^, although some uncertainties still exist^3^,^16^,^17^. According to the presently accepted scheme of the reaction (Fig. 1), hydrolysis or turnover of a single ATP molecule at each catalytic site is coupled with one revolution of the γ subunit, and the angle between the three catalytic sites differs by 120° during each catalytic phase^18^. The step size of the rotation is 120°, each coupled to the turnover of a single ATP molecule^11^. The 120° step is further divided into 80° and 40° substeps^19^,^20^. The 80° substep is triggered by ATP binding and ADP release^21^,^22^,^23^, whereas the 40° substep is triggered by ATP hydrolysis and the release of inorganic phosphate (P_i_)^15^,^18^,^20^,^22^. The angular positions of F_1_ before the 80° and 40° substeps are referred to as the ATP-binding and catalytic angles, respectively. When a β subunit's ATP-binding angle is 0° (cyan circle in Fig. 1), it executes a hydrolysis reaction after the γ subunit rotates by 200°^18^, releases ADP between 240° and 320°, and P_i_ at 320°, respectively^15^,^24^.

Using rotation assays, we recently detected the presence of a new reaction intermediate as a clear intervening pause before the 80° substep during the rotation of F_1_, by using F_1_ from the thermophilic *Bacillus* PS3 (TF_1_) at low temperatures (~9°C)^25^,^26^. The thermophilic *Bacillus* PS3 grows at 65°C, a temperature higher than that at which other species can grow. The new reaction intermediate was also observed in the rotation assay at room temperature (~28°C) using a mutant TF_1_ in which a glutamate residue at position 190 of the β-subunit (corresponding to Glu-181 of the F_1_ from *Escherichia coli* and Glu-188 of the F_1_ from bovine mitochondria) was substituted with an aspartic acid^26^,^27^. Glu-190 of the β-subunit of TF_1_ has been identified as a critical residue in ATP hydrolysis^5^,^28^,^29^,^30^, and is termed the “general base” since this residue seems to induce an in-line attack of the water molecule on the γ phosphate and initiate the hydrolysis reaction by activating the water molecule. Another single molecule study revealed that this new reaction intermediate occurs at the angle where the β subunit waits for ATP binding (0° in Fig. 1)^26^. Although this reaction has not been further characterized, the rate constant was found to be remarkably sensitive to temperature. The rate constant increased by a factor of 6–19 for every 10°C rise in temperature^25^,^26^ (Q_10_ = 6–19), which was unusually high compared to conventional Q_10_ values of around 2. In general, reactions with high Q_10_ values involve large conformational changes. Therefore, this reaction may play a key role in rotation and torque generation. Hereafter, this reaction has been referred to as the temperature-sensitive reaction (TS reaction).

To evaluate the torque generated during each step of the reaction, we recently developed a novel method to measure the equilibrium constant of the F_1_ reaction at various rotational angles^31^. Through this method, we arrested F_1_ in the transient conformation using magnetic tweezers and observed the behavior of F_1_ immediately after release from arrest. From the analysis of the behavior of F_1_, we could simultaneously determine the rate constant for each forward and reverse step of the reaction at various rotational angles. Thus, we could measure the equilibrium constant of each step of the reaction. Because the equilibrium constant is a measure of the difference in the free energy of the pre- and post-reaction states, ΔG(θ) = −*k*_B_T·ln*K*_E_(θ), the torque generated during the reaction can be estimated from the derivative of the free energy, dΔG(θ)/dθ.

In the present study, we perform a stalling experiment to elucidate how F_1_ modulates the rate and equilibrium constants of the TS reaction as a function of the rotational angle and attempt to assess its contribution in torque generation. The results will contribute to understanding the chemomechanical energy coupling of F_1_ at the resolution of the elementary reaction step.

## Results

### Temperature dependence of the rotation of the TF_1_(βE190D) mutant

We observed the rotation of the mutant TF_1_, namely, α_3_β(E190D)_3_γ, in the presence of 1 mM ATP at 18, 23, and 28°C (Fig. 2a). Between 18 and 28°C, the mutant F_1_ rotated with 80° and 40° substeps (Fig. 2b); the rate limiting steps of the 80° and 40° substeps were identified to be the TS reaction and ATP hydrolysis, respectively, in our previous study^26^. The dwell time prior to the 80° substep (TS dwell) showed a strong dependence on temperature (Fig. 2c). By fitting the histograms with exponential functions, the time constants of the TS reaction at 18, 23, and 28°C were determined to be 330, 96, and 43 ms, respectively (Fig. 2c). In contrast, the dwell time before the 40° substep (hydrolysis dwell) was not dependent on temperature and was determined to be 208 ms for 18°C, 235 ms for 23°C, and 270 ms for 28°C (Fig. 2d). These results were consistent with the results of our previous study on the TS reaction^26^.

### Manipulation of single F_1_ rotation

For manipulating the rotation of the γ subunit of F_1_, a magnetic bead (ϕ ≈ 200 nm) was attached to it and the α_3_β_3_ ring was immobilized on the glass surface. For the stalling experiments, the rotation of F_1_ was observed under conditions under which the TS dwell was lengthened enough to enable recording at approximately 1,000 fps using a mutant F_1_, α_3_β(E190D)_3_γ. As mentioned above, we can distinguish between the angular positions for the TS reaction and the hydrolysis by analyzing the TS and hydrolysis dwell times (Figs. 2c, 2d). When F_1_ paused for the TS reaction, we turned on the magnetic tweezers to arrest F_1_ at the target angle (Fig. 3a). After the set period had elapsed, we turned off the magnetic tweezers and released F_1_ from the arrest. Following release, F_1_ showed one of two behaviors: rotating directly forward to the next catalytic angle (red in Fig. 3b), i.e., skipping the pause at the original ATP-binding angle, or returning to the original ATP-binding angle (blue in Fig. 3b) without exception. Forward rotation of F_1_ implied that it had completed the TS reaction and exerted a torque on the magnetic beads; backward rotation of F_1_ meant that it had not completed the TS reaction because it did not catalyze the reaction and hence could not generate a torque. These behaviors of F_1_ are hereafter referred to as “ON” (forward rotation) and “OFF” (backward rotation), respectively. Using the above-mentioned methodology, we conducted the stalling experiments in the angle range of ±50°, where the standard deviation of the arrested angle was 5.8°. The following sections discuss the analysis of the probability (*P*_ON_) of ON events against the total trials.

### Angular dependence of the kinetic parameters of the TS reaction

**Figure 1:**
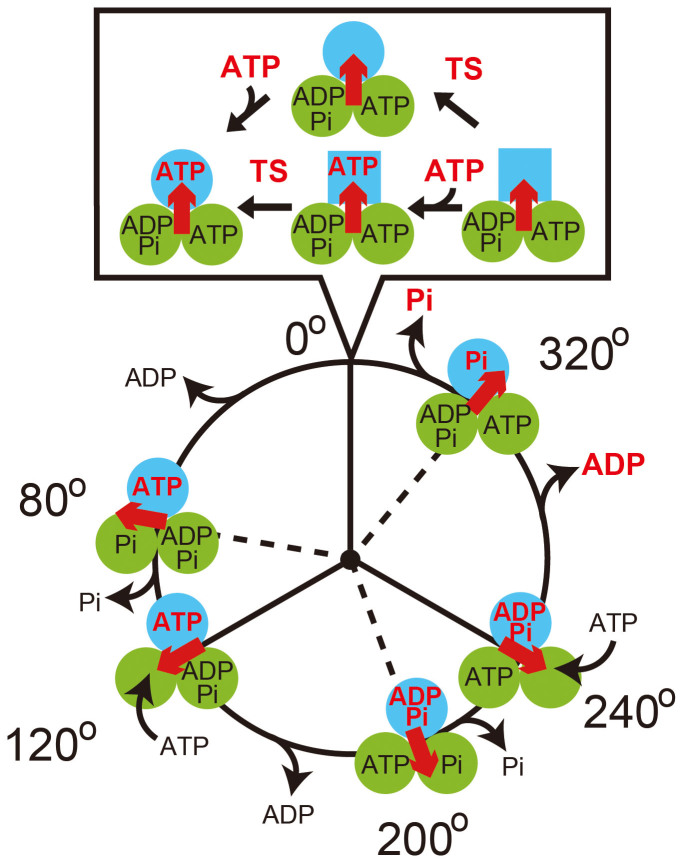
Reaction scheme of F_1_ The circles and red arrows represent the catalytic states of the β subunits and the angular positions of the γ subunit, respectively. Each β subunit hydrolyzes a single molecule of ATP during one turn of γ, whereas three β subunits differ the reaction phase by 120°. The catalytic state of the top β subunit (cyan) has been indicated for clarity. ATP binding, TS reaction, hydrolysis, ADP release, and P_i_ release occur at 0°, 0°, 200°, 240°, and 320°, respectively.

**Figure 2:**
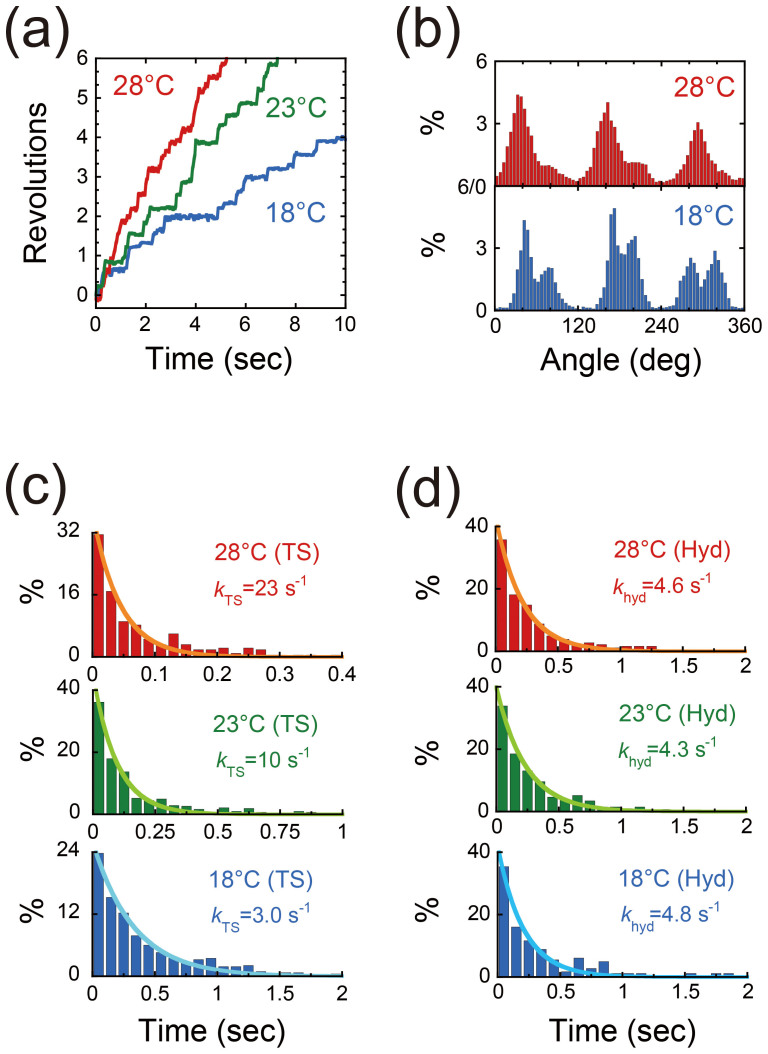
Rotation of mutant F_1_ (α_3_β(E190D)_3_γ) at various temperatures. (a). Time course of rotation of the mutant F_1_ in the presence of 1 mM ATP at 18 (blue), 23 (green), and 28°C (red). (b). Histograms of the angular position during rotation as calculated from Fig. 2a. (c, d). Histograms of the dwell time of the pause prior to the 80° substep (TS dwell) or the 40° substep (hydrolysis dwell). Curves were plotted using a single-order reaction scheme. y = *C*·exp(−*k*t), where *k*_TS_^on^(18°C) = 3.0 s^−1^, *k*_TS_^on^(23°C) = 10 s^−1^, *k*_TS_^on^(28°C) = 23 s^−1^, *k*_hyd_(18°C) = 4.8 s^−1^, *k*_hyd_(23°C) = 4.3 s^−1^, and *k*_hyd_(28°C) = 4.6 s^−1^.

**Figure 3:**
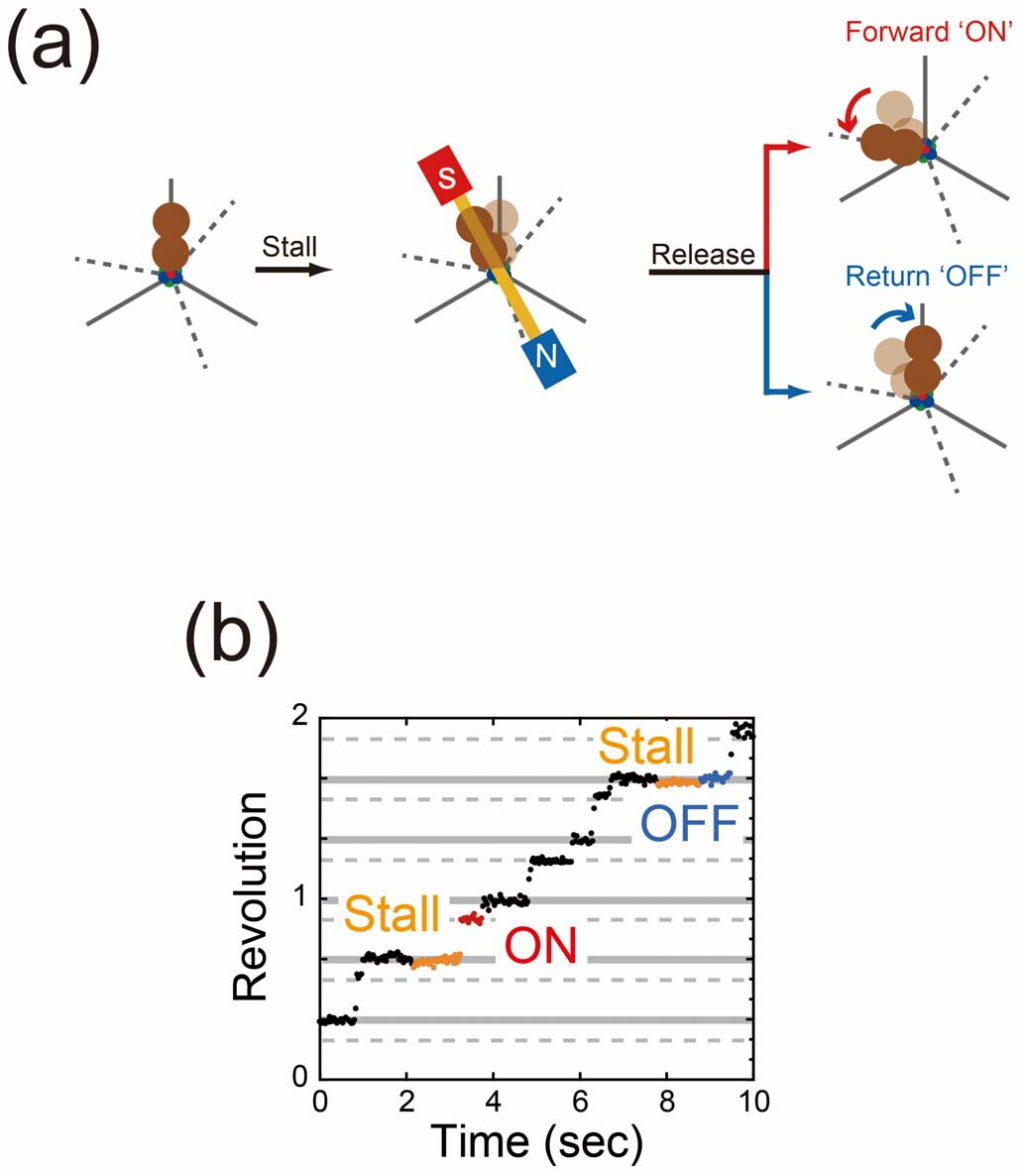
Single-molecule manipulation of F_1_. (a). Schematic image of the manipulation procedures. The gray solid and dashed lines represent the ATP-binding and catalytic angles, respectively. When F_1_ paused due to TS dwell at the ATP-binding angle, the magnetic tweezers were switched on to stall F_1_ at the target angle and then turned off to release the motor after the set period had elapsed. A released F_1_ showed forward (ON) or backward (OFF) rotation with respect to the original ATP binding angle. The behavior of F_1 _indicated whether the TS reaction was completed (in case of ON) or not (in case of OFF). (b). Examples of stalling experiments for the TS reaction at 18°C. During a pause, F_1_ was stalled at −6.6° from the original pausing angle for 1.0 s and then released. After being released, F_1_ rotated to the next catalytic angle without any backward rotation, indicating that the TS reaction had been completed by F_1_ upon release (red). When F_1_ was stalled at −9.1° for 1.0 s, it rotated back to its original pausing angle, implying that the TS reaction had not been completed (blue).

**Figure 4:**
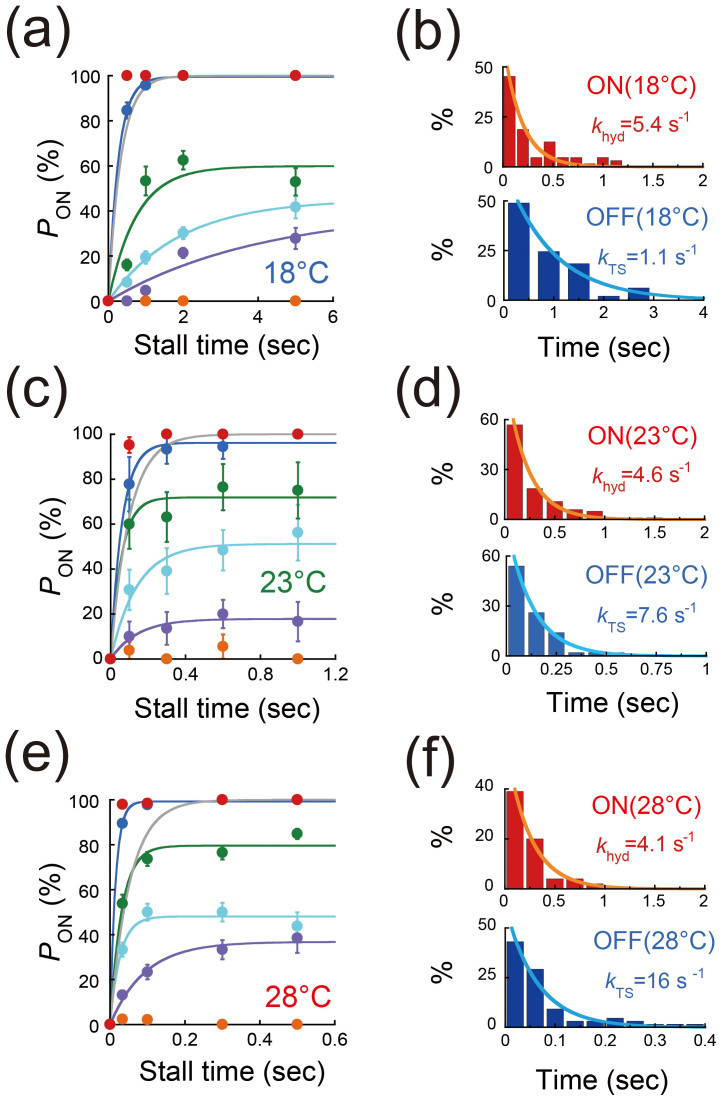
Angular dependence of the TS reaction by the mutant F_1_ (α_3_β(E190D)_3_γ). (a, c, e). Time course of *P*_ON_ of F_1_ (α_3_β(E190D)_3_γ) in the presence of 1 mM ATP at 18, 23, and 28°C, after stalling at −30° (orange), −10° (purple), 0° (cyan), +10° (green), +30° (blue), and +50° (red). The gray lines represent the time courses for freely rotating F_1_s. *k*_TS_^on^ and *k*_TS_^off^ were determined by fitting a single exponential function: *P*_ON_ = (*k*_TS_^on^/(*k*_TS_^on^ + *k*_TS_^off^))·[1 − exp (−(*k*_TS_^on^ + *k*_TS_^off^)·t)], according to the reversible reaction scheme, F_1_ 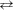 F_1_*. Each data point was obtained from 13–63 trials using 4 molecules. The error in *P*_ON_ is represented as 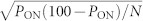, where *N* is the number of trials for each stall measurement. (b, d, f). Histograms of dwell times immediately after the stalling at 18, 23, and 28°C. Top panels represent the dwell time to conduct another 40° step (hydrolysis dwell) after the ON event (red points in Fig. 3b). Bottom panels represent the dwell time to conduct spontaneously an 80° step after an OFF event (blue points in Fig. 3b). Curves were obtained by fitting the data to a single-order reaction scheme. y = *C*·exp(−*k*t), where *k*_hyd_(18°C) = 5.4 s^−1^, *k*_TS_^on^(18°C) = 1.1 s^−1^, *k*_hyd_(23°C) = 4.6 s^−1^, *k*_TS_^on^(23°C) = 7.6 s^−1^, *k*_hyd_(28°C) = 4.1 s^−1^, and *k*_TS_^on^(28°C) = 16 s^−1^.

Using the mutant F_1_, experiments were conducted at 18°C, where the TS dwell time was 330 ms (Fig. 2c). Fig. 4a shows *P*_ON_ plotted against the stall time. *P*_ON_ increased with both the stall angle and the stall time (Fig. 4a), which is similar to our previous observation of ATP binding to wild-type F_1_^31^. In addition, *P*_ON_ did not always saturate to 100% but converged to a certain value, e.g., 60% for +10° stall (green line in Fig. 4a). These observations imply that the TS reaction is reversible, and that reverse reaction also occurs during stalling. Therefore, the plateau level indicates the equilibrium level between the pre- and post-TS reaction states. To confirm the reversibility, we analyzed the behaviors immediately after the OFF events; i.e., dwell times to spontaneously conduct 80° steps (dwell times at 0° in Fig. 1) immediately after the OFF events (blue points in Fig. 3b). Here, to avoid including data from before the equilibrium, only experiments with longer stalling times, in which *P*_ON_ achieved a plateau were analyzed. The dwell time histogram obtained from all the stall angles showed a single exponential decay, providing a rate constant of 1.1 s^−1^ (bottom panel in Fig. 4b), which corresponded to that obtained for freely rotating F_1_s, which were not manipulated with magnetic tweezers (Fig. 2c). This correspondence excluded the possibility of any unexpected inactivation occurring during the stalling that might compete with the TS reaction. We also plotted a histogram of the dwell times to conduct 40° steps (dwell times at 80° in Fig. 1) after the ON events (red points in Fig. 3b). This histogram (top panel in Fig. 4b) was also in good agreement with that obtained for freely rotating F_1_s (Fig. 2d), confirming that the manipulation did not alter the kinetic properties of F_1_.

**Figure 5:**
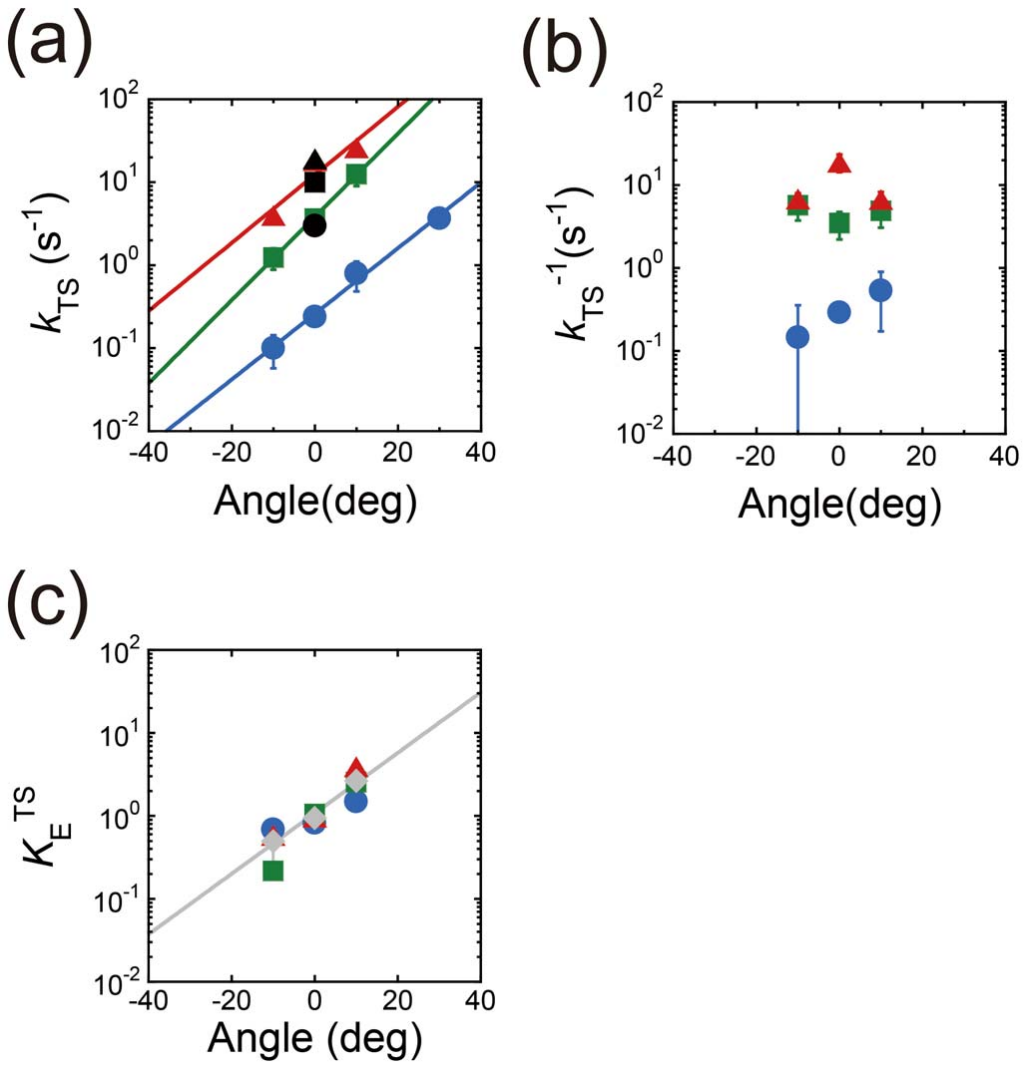
Angular dependence of kinetic parameters. Angular dependence of *k*_TS_^on^, *k*_TS_^off^, and *K*_E_^TS^ Blue circle, green square, and red triangle symbols represent the values for 18, 23, and 28°C, as determined from Figs. 4a, 4c, and 4e, respectively. In (a), the black symbols represent *k*_TS_^on^ obtained from the freely rotating F_1_s (Fig 2c). In (c), the gray symbols represent the average of *K*_E_^TS^ for 18, 23, and 28°C.

By fitting the time courses of *P*_ON_ based on a reversible reaction scheme, F_1_ 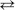 F_1_*, the rate constants of the TS reaction and its reverse reaction, *k*_TS_^on^(18°C) and *k*_TS_^off^(18°C), were determined for each stall angle (Figs. 5a and 5b). *k*_TS_^on^(18°C) increased exponentially with the stall angle by approximately 6.2 fold per 20°, which was double that reported previously for ATP binding^31^. *k*_TS_^on^(18°C) at ±0° was evidently slower than that determined for freely rotating F_1_s. This phenomenon, which is similar to the ATP-binding step, is attributed to thermal agitated rotary fluctuation that occasionally pushes γ forward, accelerating the TS reaction. In contrast, *k*_TS_^off^(18°C) was almost constant at approximately 0.3 s^−1^. Therefore, the equilibrium constant of the TS reaction [*k*_TS_^on^(18°C)/*k*_TS_^off^ (18°C) = *K*_E_^TS^(18°C)] increased 2.2 times from −10° to + 10° (blue points in Fig. 5c), which is a steeper angle dependence than that observed for ATP hydrolysis in the previous study^31^.

To confirm the strong angle dependence of the TS reaction under a different condition, the stalling experiments were also conducted at 23 and 28°C, where the time constants of the TS reaction were 96 and 43 ms, respectively (Fig. 2c). The time courses of *P*_ON_ showed the same tendency as that observed at 18°C (Figs. 4c, 4e). The reversibility of the TS reaction at 23 and 28°C was also confirmed from the analysis of the dwell time after arrest (Figs. 4d and 4f). The rate and equilibrium constants were determined as mentioned above (Figs. 5a, 5b, and 5c). The equilibrium constants determined for the TS reaction at 23 and 28°C showed essentially the same angle dependence as that at 18°C (red and green points in Fig. 5c). Thus, the strong angle dependence of the TS reaction is inherent in F_1_, regardless of the temperature.

### Rotational energy potential

**Figure 6:**
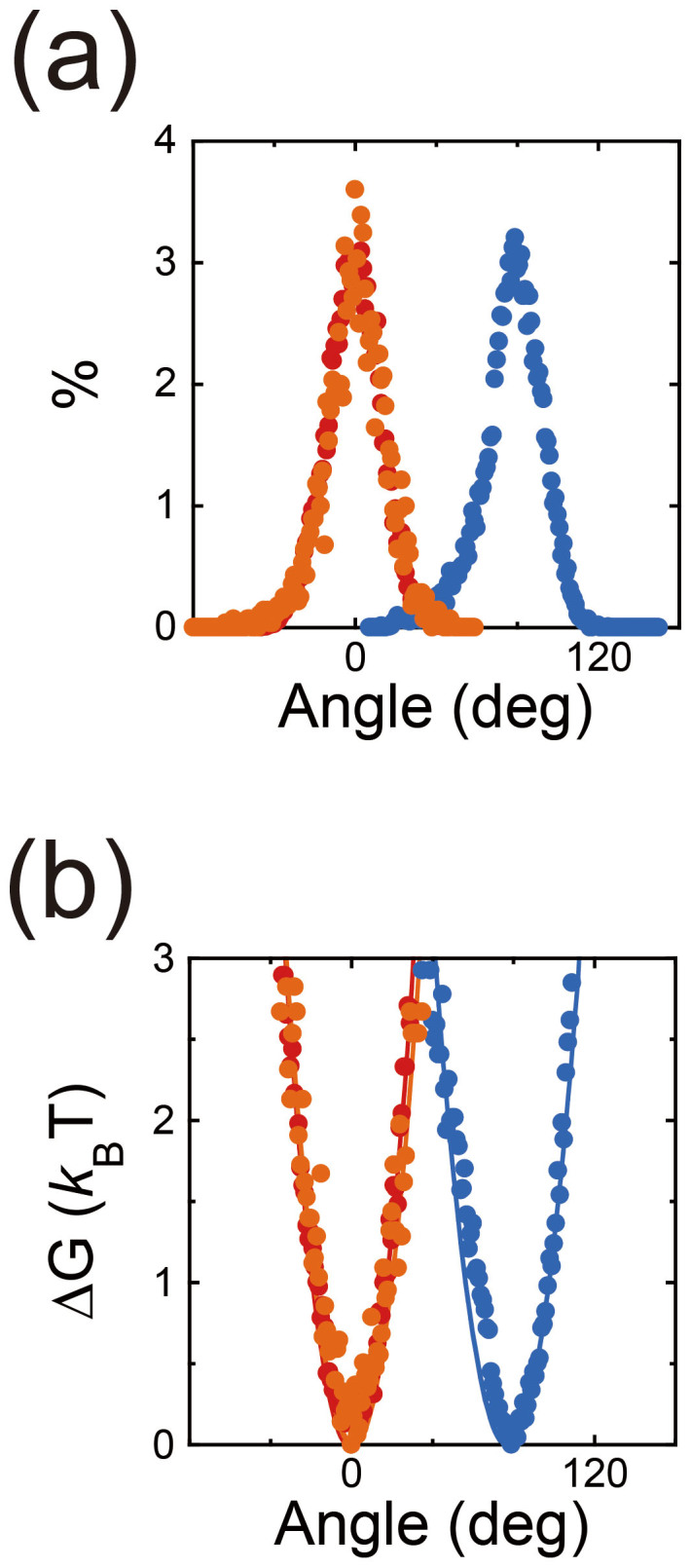
Rotational energy potential. (a). Probability distributions of angular positions during the dwell. Red and blue points represent our previous results for the ATP binding dwell of wild-type F_1_ and the hydrolysis dwell of mutant F_1_, α_3_β(E190D)_3_γ^31^. Orange points represent the experimental result for the TS dwell of mutant F_1_ measured in this study. The probability distribution was derived from three molecules. (b). Rotational energy potentials determined from probability distribution according to the Boltzman's law, ΔG = −*k*_B_T·ln(*P*/*P*_o_). The potentials determined were fitted to the harmonic function ΔG = 1/2·*κ*·θ^2^, where *κ* is the torsion stiffness. Stiffness values determined were 80, 75, and 64 pN·nm for the ATP binding of wild-type F_1_, the TS reaction, and the hydrolysis of mutant F_1_, respectively.

We examined the rotational energy potential during TS dwell, i.e., the waiting state for the TS reaction. The probability distribution of γ-orientation during the TS dwell of mutant F_1_, α_3_β(E190D)_3_γ, was measured (orange points in Fig. 6a). The probability distributions obtained were then transformed into the rotational energy potential according to the Boltzmann's Law, ΔG = −*k*_B_T·ln(*P*/*P*_o_) (orange points in Fig. 6b). The potential determined was fitted to the harmonic function, ΔG = 1/2·*κ*·θ^2^, where *κ* is the torsion stiffness. The determined value of stiffness was 75 pN·nm, which was similar to the values for ATP binding of wild-type F_1_ and hydrolysis of mutant F_1_, α_3_β(E190D)_3_γ, determined in the previous study^31^ (red and blue points in Fig. 6b). This result suggested that the magnitude of rotational energy potential in the pre-reaction state did not depend on individual reaction steps.

## Discussion

**Figure 7:**
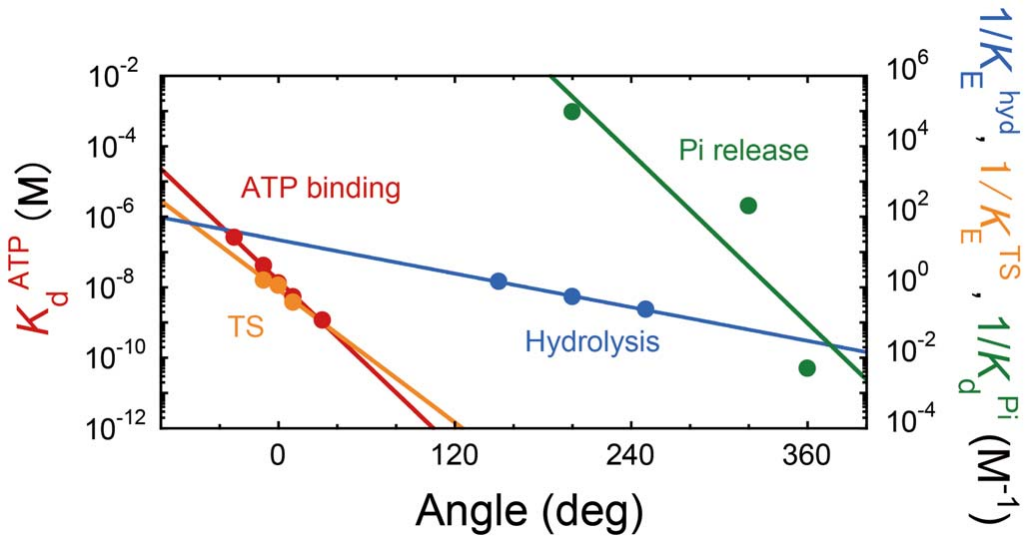
Modulation of equilibrium constants upon γ rotation. Modulation of equilibrium constants upon rotation. All data points are plotted along the reaction scheme for one β subunit (Fig. 1), where the angles for ATP binding, TS reaction, hydrolysis, and P_i_ release are assigned as 0°, 0°, 200°, and 320°, respectively. Red, blue, and green symbols represent the dissociation constant of ATP (*K*_d_^ATP^), the inverse values of the equilibrium constant of ATP hydrolysis (1/*K*_E_^Hyd^), and the dissociation constant of P_i_ (1/*K*_d_^Pi^), determined in the previous study^15^,^22^,^31^. Orange symbols represent the inverse values of the equilibrium constant of the TS reaction (1/*K*_E_^TS^), determined in this study.

The equilibrium constant of the TS reaction determined in this study, as well as those of the other reaction steps, that is, ATP binding, hydrolysis, and P_i_ release, determined in our previous studies^15^,^31^, are shown in Fig. 7. Data points are plotted in the angular diagram of the reaction scheme for one β subunit (Fig. 1), where the pause angles for ATP binding, TS reaction, hydrolysis, and P_i_ release were assigned as 0°, 0°, 200° and 320°, respectively. The magnitude of rotational torque (*N*) is determined by the slope of the rotational energy potential in the post-reaction state, d*U*_post_(θ)/dθ^31^,^32^. It is very difficult to measure the rotational potential directly in the post-reaction state, *U*_post_(θ). Therefore, in our previous studies, we had estimated the torque generated during each reaction step from the angular dependence of the reverse reaction rate, Δ*G*_1_(θ) = −*k*_B_T·[ln*k*^−1^(θ)]^31^,^32^, which is a measure of the energy difference between the transition state and the post-reaction state, Δ*G*_1_(θ) = *U*_post_(θ) − *U*_TS_(θ). When we assume that the energy level at the transition state, *U*_TS_(θ), is a constant, and does not depend on the rotational angle, the derivative of the energy difference responds to the slope of the potential in the post-reaction state (equivalent to the torque), dΔ*G*_1_(θ)/dθ = d*U*_post_(θ)/dθ. Therefore, we previously estimated the torque generation from the reverse reaction rate based on this assumption with respect to the energy level for the transition state^31^,^32^, which has not been verified experimentally so far. In this study, we used the angle dependence of the equilibrium constant, *K*_E_(θ), which is a more robust approach to estimate the torque generation. The free energy difference between the pre- and post-reaction states can be determined from the angle dependence of the equilibrium constant; Δ*G*_2_(θ) = *U*_post_(θ) – *U*_pre_(θ) = *k*_B_T·[ln(*K*_E_(θ))]. Because *U*_pre_(θ) was not affected by the elastic component located on the transmission line to the beads^9^, and was almost the same for each reaction step (Fig. 6), the derivative of the free energy difference may be regarded as a measure of the slope of the rotational potential in the post-reaction state (equivalent to the torque), dΔ*G*_2_(θ)/dθ ≈ d*U*_post_(θ)/dθ. Therefore, the slopes of the equilibrium constants in a semi-log plot (Fig. 7) reflect the magnitude of torque generated upon each reaction step. The estimation shows that the TS reaction has a slope similar to those of ATP binding and P_i_ release and a steeper slope than that of ATP hydrolysis. This suggests that the contribution of the TS reaction to torque generation is similar to those of ATP binding and P_i_ release and is much higher than that of ATP hydrolysis, i.e., the TS reaction contributes significantly to the torque generation of F_1_.

Considering the extremely high temperature dependence of the TS reaction, this reaction may involve a large-scale conformational rearrangement of the catalytic β-subunit when the γ is oriented to the angle for ATP binding. Recent single-molecule studies have revealed that the *C*-terminal region of the β subunit shows a large-scale conformational change at around 0°^5^,^33^, which contributes to generating half of the rotational torque, that is, approximately 20 pN·nm rad^−1^^34^,^35^. Our experimental results suggest that the TS reaction contributes greatly to torque generation at around 0°. Therefore, it is probable that the TS reaction is somehow related to the large-conformational change in the *C*-terminal region of the β subunit at 0°; however, there has been no direct verification so far. To identify the TS reaction, we hope to visualize simultaneously the conformational change in the β subunit and the rotational motion at the temperature, where F_1_ shows a distinctive pause due to the TS reaction.

Improper ATP hydrolysis due to an alternative catalytic pathway^27^ may be another possible reason for the occurrence of the TS reaction. According to this mechanism, P_i_ release in the β-subunit at the 320° state (cyan circle at 320° in Fig. 1) may drive the rotation of the 40° substep from 320° to 360° ( = 0°) without hydrolyzing ATP in another β-subunit at the 200° state (left green circle at 320° in Fig. 1). This may cause the dwell at 0° for waiting the ATP hydrolysis to occur in the aforementioned β-subunit at the 240° state (left green circle at 0° in Fig. 1). In our experimental data, the angle dependence of the rate constants of the TS reaction and its reverse reaction (Fig. 5) was similar to those of ATP hydrolysis and synthesis^31^. The forward reaction rate was accelerated towards the anticlockwise direction, while the reverse reaction rate was almost constant and did not depend on the rotary angle, although the slopes of angle dependence are different from each other. Therefore, our result suggests that the TS reaction might occur due to improper ATP hydrolysis at the 240° state (left green circle at 0° in Fig. 1). Simultaneous monitoring of the catalytic events, i.e., ATP binding, hydrolysis, and products release, with the rotational motion will provide insights into this mechanism.

Using single-molecule manipulations, we measured the rate and equilibrium constants of F_1_ in the transient conformational states, which could not be obtained in the conventional single molecule assay. Moreover, from the equilibrium constants determined by single-molecule manipulations, we evaluated the force generated during the elementary reaction steps. Thus, single-molecule manipulation is a powerful tool for understanding the chemomechanical energy coupling mechanism and holds promise for understanding the functioning of other molecular machines.

## Methods

### Rotation assay

The mutant form of F_1_ from thermophilic *Bacillus* PS3 (TF_1_), α_3_β(E190D)_3_γ, was prepared as described previously^36^. To visualize the rotation of F_1_, the stator region (α_3_β_3_ subunits) was fixed to a glass surface, and magnetic beads (ϕ approximately 0.3 μm; Seradyn, USA) were attached to the rotor (γ subunit), as the probe for monitoring rotation and for further manipulation. The rotation assay was carried out in a 50 mM MOPS-KOH (pH 7.0) buffer containing 50 mM KCl, 5 mM MgCl_2_, and 1 mM ATP. Rotating beads were observed under a phase-contrast microscope (IX-70 or IX-71; Olympus, Japan) with a 100× objective lens. The temperature in the room was controlled with a room air conditioner and monitored with a thermometer located on the sample stage of the microscope. The precision of the temperature control was ±1°C.

### Manipulation with magnetic tweezers

The stage of the microscope was equipped with magnetic tweezers that could be controlled with the custom-made software (Celery, Library, Japan). The rotation of the bead was simultaneously recorded at 30 and 1,000–3,000 frames/s (FC300M, Takex; FASTCAM 1024PCI-SE, Photoron, Japan). Images were stored in the HDD of a computer as AVI files and analyzed using the custom-made software.

## Author Contributions

R. W. designed and performed the experiments and analyzed the data; H. N. designed the experiments, built the whole story, and wrote the paper with R.W.
